# Bilateral Spontaneous Pneumothorax in a COVID-19 and HIV-Positive Patient: A Case Report

**DOI:** 10.3389/fmed.2021.698268

**Published:** 2021-12-16

**Authors:** Young Min Cho, Sara Guevara, Judith Aronsohn, James M. Mumford, Linda Shore-Lesserson, Santiago J. Miyara, Martin Herrera, Claudia Kirsch, Christine N. Metz, Stefanos Zafeiropoulos, Dimitrios Giannis, Alexia McCann-Molmenti, Kei Hayashida, Koichiro Shinozaki, Muhammad Shoaib, Rishabh C. Choudhary, Gabriel I. Aranalde, Lance B. Becker, Ernesto P. Molmenti, James Kruer, Anthony Hatoum

**Affiliations:** ^1^Department of Internal Medicine, Northeast Georgia Medical Center, Gainesville, GA, United States; ^2^Department of Surgery, North Shore University Hospital, Manhasset, NY, United States; ^3^Department of Anesthesiology, North Shore University Hospital, Manhasset, NY, United States; ^4^Donald and Barbara Zucker School of Medicine at Hofstra/Northwell, Hempstead, NY, United States; ^5^Department of Family Medicine, Glen Cove Hospital, Glen Cove, NY, United States; ^6^Elmezzi Graduate School of Molecular Medicine, Manhasset, NY, United States; ^7^Feinstein Institutes for Medical Research, Manhasset, NY, United States; ^8^Department of Emergency Medicine, North Shore University Hospital, Manhasset, NY, United States

**Keywords:** COVID-19, SARS-CoV-2, spontaneous pneumothorax, tension pneumothorax, bilateral pneumothorax, HIV/AIDS, HAART

## Abstract

This case report describes a 60 year-old Black-American male with a past medical history of human immunodeficiency virus (HIV) infection and hyperthyroidism, who suffered a bilateral spontaneous pneumothorax (SP) in the setting of coronavirus disease 2019 (COVID-19) pneumonia. SP is a well-established complication in HIV-positive patients and only recently has been associated with severe acute respiratory syndrome coronavirus-2 (SARS-CoV-2) infection. While HIV and COVID-19 infections have been independently linked with increased risk of SP development, it is unknown if both infections interact in a synergistic fashion to exacerbate SP risk. According to the Centers for Disease Control and Prevention (CDC), patients living with HIV have a higher risk of developing severe COVID-19 infection and the mechanism remains to be elucidated. To the best of our knowledge, this is the first report of a HIV-positive patient, who in the setting of SARS-CoV-2 infection, developed bilateral apical spontaneous pneumothorax and was later found to have a left lower lobe tension pneumothorax. This case highlights the importance of considering SP on the differential diagnosis when HIV-positive patients suddenly develop respiratory distress in the setting of SARS-CoV-2 infection.

## Introduction

Spontaneous pneumothorax (SP) is defined as a pathological collection of gas in the pleural space without traumatic mechanisms such as mechanical or iatrogenic trauma (1). SP usually occurs in tall, slim teenagers and men between the ages of 10 and 30 years old (2). Smoking increases the risk of SP in a dose-dependent manner, with an average of 20-fold risk increase (3, 4). Symptomatically, most patients present with ipsilateral pleuritic chest pain and dyspnea. Chest pain severity can range from mild to severe, is frequently described as “sharp,” and may resolve within 24 h without intervention, unlike a tension pneumothorax which may require intervention, such as needle decompression and chest tube placement. Tachycardia is the most common vital sign abnormality (5).

SP is a well-established complication in HIV-infected patients (6–8). On average, 5% of acquired immunodeficiency syndrome (AIDS) patients develop SP at some point during the disease course (9). The mechanisms linking HIV infection with SP are unknown, however, *Pneumocystis jirovecii* (PCP) infection has been deemed as the most common etiology (10). Cystic lung lesions have also been reported with HIV-positive patients in the setting of PCP pneumonia, and are considered potential precursors to SP (11).

SP is described as a complication of coronavirus disease 2019 (COVID-19) pneumonia (12, 13). The incidence of SP in patients with COVID-19 is not well-established, however, in an observational study published by Chen et al. 1% of the patients had SP (14). In a second study by Yang et al., in a series of 92 deceased COVID-19 patients, only one presented with SP, causing his death 5 days after symptom onset (15). In a third study by Zantah et al. a review of 3,000 patients demonstrated an incidence of 0.66%. Why SP occurs during COVID-19 infection is still undetermined, however, it has been hypothesized that the severe acute respiratory syndrome coronavirus-2 (SARS-CoV-2) causes cystic and fibrotic degeneration in the lung parenchyma, ultimately leading to alveolar tearing (16). Furthermore, mechanical ventilation and continuous cough associated with COVID-19, results in increased intrapulmonary pressure, which is a known risk factor for SP development (17, 18).

In this case study, we describe the clinical course of a 60-year-old Black-American male with a past medical history of HIV infection [controlled by treatment with highly active antiretroviral therapy (HAART)], HIV-associated encephalopathy, and hyperthyroidism. To date, this is the first published case report of an HIV-positive patient who in the setting of COVID-19 infection, developed bilateral spontaneous pneumothorax, and later developed a left side tension pneumothorax.

## Case Presentation

A 60-year-old Black male with a past medical history of HIV infection with a CD_4_ T cell count of 351 cells/mm^3^ on HAART, HIV encephalopathy, hyperthyroidism, BMI 18.2 kg/m^2^ (height 1.7 cm and weight 52 Kg) with no previous history of lung disease such as chronic obstructive pulmonary disease (COPD) or opportunistic disease documented and no active smoking or respiratory disease presented to the hospital due to worsening confusion, cough, and generalized weakness. Initial vital signs included temperature of 36.9°C, blood pressure (BP) 95/77 mmHg, heart rate (HR) 110 beats/min, respiratory rate (RR) 18 breaths/min, and oxygen saturation of 97% on room air.

On physical exam, the patient was noted to be frail, tachycardic, and demonstrated appreciable scattered rhonchi on auscultation. Laboratory testing was remarkable for leukocytosis 11.6 K/μL (normal 4.8–10.8 K/μL), D-Dimer 4 μg/ml (normal <0.4 μg/ml), fibrinogen 980 mg/dL (normal 196–493 mg/dL), erythrocyte sedimentation rate (ESR) 76 mm/h (normal 0–20 mm/h), C-reactive protein (CRP) 26 mg/L (normal 0–0.6 mg/L), lactate dehydrogenase (LDH) 322 u/L (normal 140–280 u/L), CD_4_ T cell count 351 cells/mm^3^ (normal 500–1,500 cells/mm^3^), CD_4_% of 23%, CD_8_ T cell count 258 cells/mm^3^ (150–1,000 cells/mm^3^), undetectable HIV viral load, procalcitonin 0.88 ng/ml (normal <0.1 ng/ml), and serum (1,3)—Beta-D-glucan <31 pg/mL (negative) on early admission. HAART treatment with dolutegravir, emtricitabine, and tenofovir alafenamide was continued throughout the admission without interruptions. The chest x-ray showed diffuse parenchymal infiltrates bilaterally with some sparing of the right upper lobe. SARS-CoV-2 PCR test resulted positive, and he was started on hydroxychloroquine therapy, which was the standard of care at that time.

**Figure 1:**
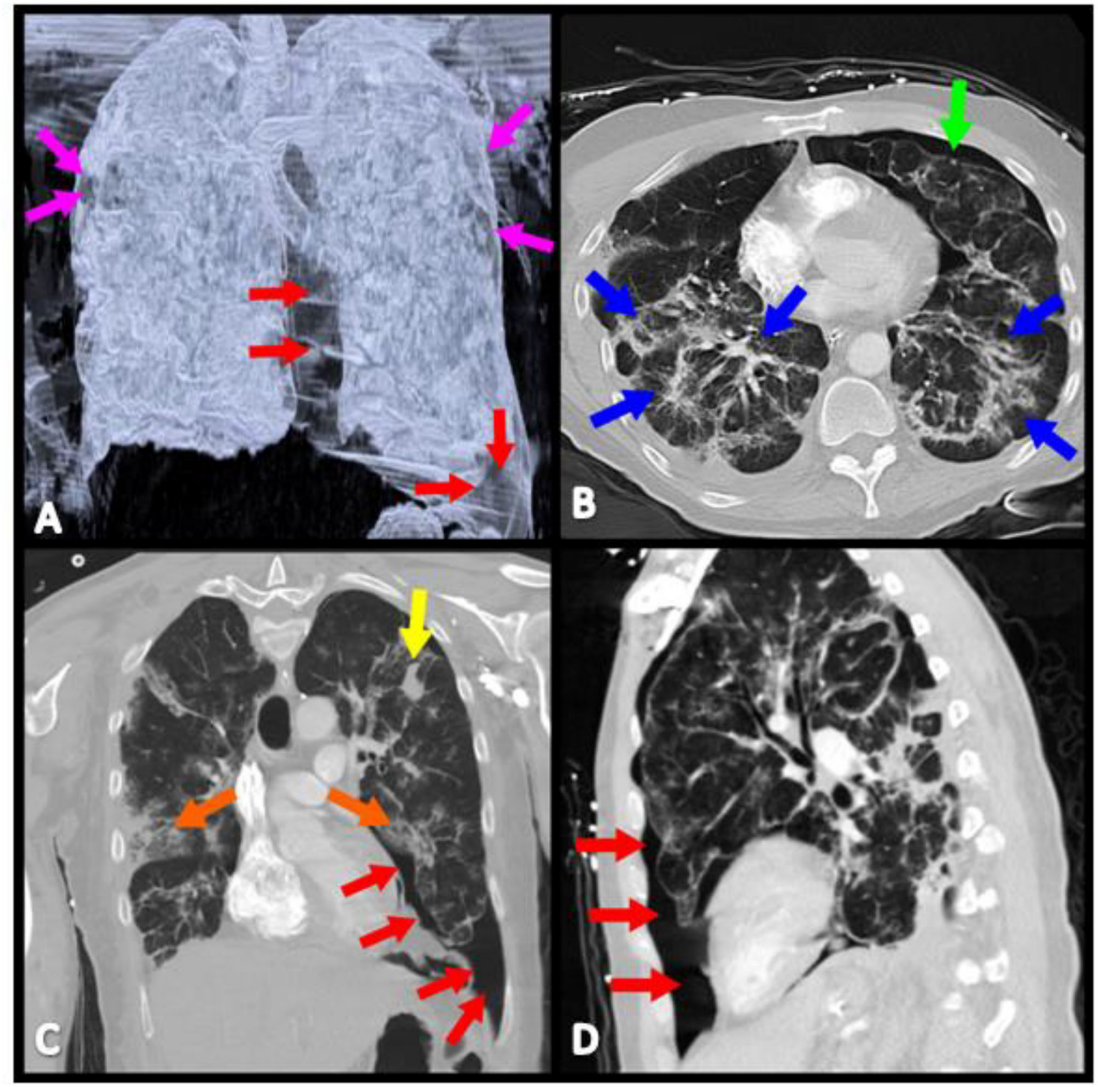
Contrasted CT scan reveals bilateral lung disease, characterized by: **(A)** Bilateral pneumothoraces (magenta, red, and green arrows). **(B)** Interstitial, diffuse infiltrates (blue arrows). **(C)** Isolated areas of consolidation (orange and yellow arrows). **(D)** Left lung pneumothorax has its largest prominence in the inferior costophrenic sulcus (red arrows).

**Figure 2:**
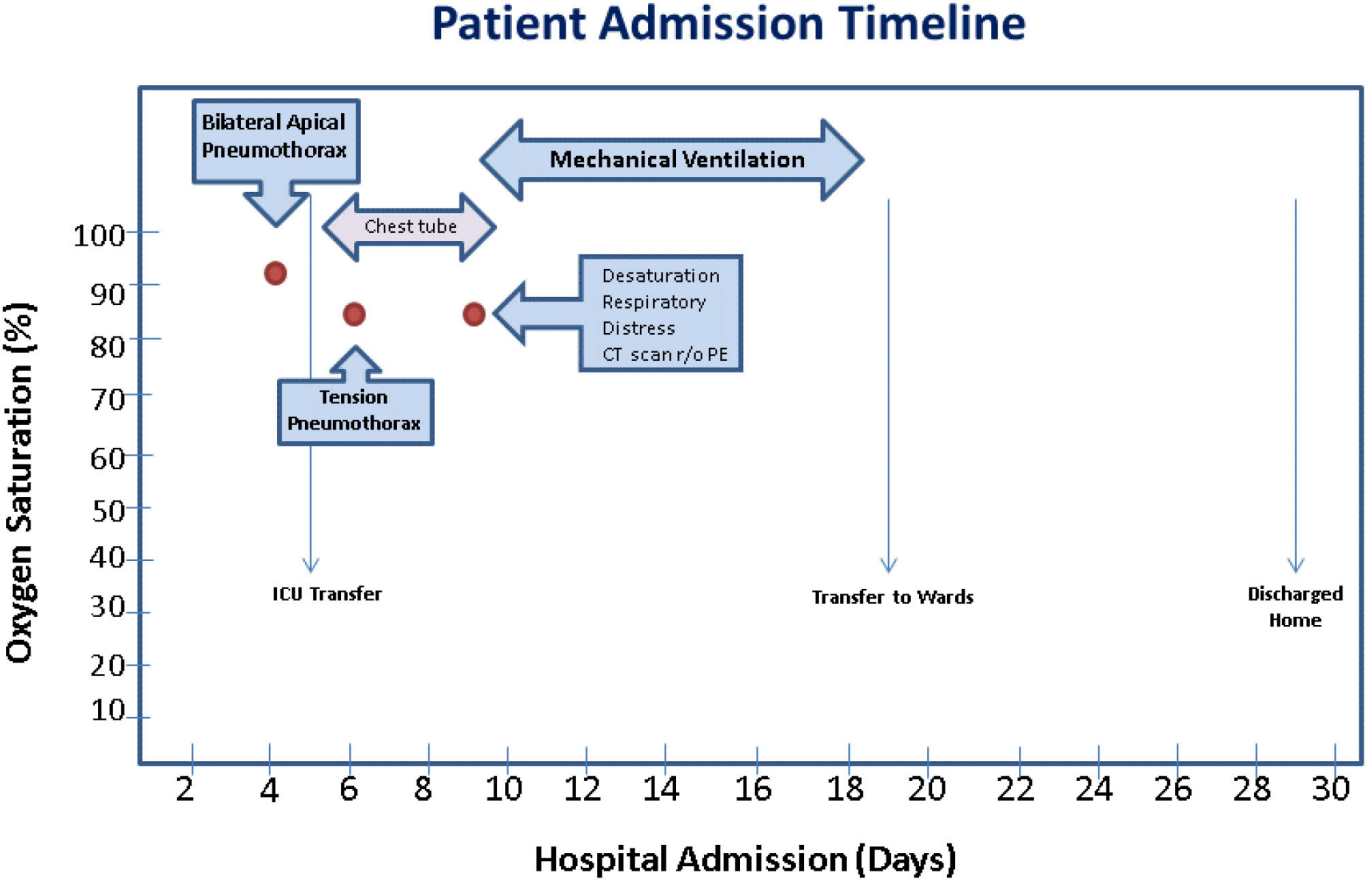
Admission Timeline of a 60-year-old Black male with a past medical history of HIV infection with a CD_4_ T cell count of 351 cells/mm^3^ on HAART, HIV encephalopathy, hyperthyroidism, BMI 18.2 kg/m^2^, and no active smoking or respiratory disease presented to the hospital due to worsening confusion, cough, and generalized weakness, diagnosed with COVID-19 pneumonia and bilateral pneumothoraces.

During the hospital course, febrile episodes, typical of SARS-CoV-2 infection, over 38.4°C were noted. On the fourth day of admission, the patient had worsening dyspnea prompting repeat chest x-ray, which revealed bilateral 20% apical pneumothoraces without tension (Figure 1). His respiratory status continued to decline with worsening tachypnea and he was transferred to the intensive care unit with oxygen therapy via non-rebreather mask at a flow rate of 12 L/min (Figure 2). After ICU transfer, the patient's oxygen saturation dropped to 85%, with BP of 107/77 mmHg, RR range of 31–37 breaths/min, HR of 115 beats/min, and was found to have a left-sided tension pneumothorax. Subsequently, a 20 French (FR) of chest tube was placed on fourth mid-axillary line and connected securely with tape to a chest drainage system after verified by chest x-ray; however the patient's oxygen requirements continued to increase. Arterial blood gas (ABG) showed a pH 7.49, partial pressure of carbon dioxide (PaCO2) 36.8 mmHg, partial pressure of oxygen (PaO2) 64 mmHg. Patient was intubated and mechanical ventilator started on volume control/assisted control (VC/AC), RR 12, tidal volume (TV) 262 ml (6 ml/kg), peak-end-expiratory pressure (PEEP) 8 cmH_2_o, and fractioned of inspired oxygen (FiO2) 100%. On the ninth day of admission, the patient's oxygen saturation declined again and pulmonary CT and CTA was performed to rule out another etiology, such as a pulmonary embolism (PE). CT confirmed the presence of a left pneumothorax with 10% of hemithorax volume in size mostly in the inferior costophrenic sulcus and PE was ruled out (Figure 1). On hospital day 18, after remaining hemodynamically stable, pneumothorax was reabsorbed and the patient was extubated and transferred to the inpatient medical floor, and 11 days later was discharged in stable condition breathing on room air.

## Discussion

Spontaneous pneumothorax is a potentially life-threatening event. It is commonly present with associated conditions such as COPD, cystic fibrosis, status asthmaticus, pneumonia, and lung cancer (19). A systematic review of 919 patients infected with SARS-CoV-2 by Salehi et al. reported very few cases of pneumothorax which was described as an uncommon but possible progression of the disease (19). A single retrospective study in Wuhan, China by Yang et al. reported 2% pneumothorax occurrence in patients with SARS-CoV-2 (20). A recent publication by Spiro et al. reported two cases of spontaneous pneumothorax (21). These two cases added to the body of literature that spontaneous pneumothorax is possible without any associated preexisting conditions for pneumothorax other than being positive for SARS-CoV-2. In a multicentric study published by Miró et al. 40 patients (0.05%) were diagnosed with SP from a cohort of 71,904 patients; when those patients were independently compared with COVID-19 non-SP and non-COVID-19 SP controls, it was found that SP in COVID-19 was associated with worse outcomes (22).

The presenting patient had no history of respiratory disease, no active smoking history in the last 30 years, and had not received mechanical ventilation support prior to presentation, which are known risk factors for potential development of pneumothorax complications. Interestingly, as described by Härter et al. people living with HIV, due to defective cellular immunity, may paradoxically be protected from cytokine dysregulation (23). However, our patient was on appropriate HAART, with a CD4:CD8 ratio >1, and an undetectable viral load, likely allowing for immune system inflammatory response. Although the patient may have some underlying lung disease due to his history of HIV, the patient was not aware of any disease, and there was no documentation in his chart. There is a potential association between the elevated inflammatory markers and diffuse lung injury, therefore increasing the risk of spontaneous pneumothorax (24). The presenting patient had elevated LDH, ESR, CRP, and D-dimer in the setting of SARS-CoV-2 infection. Notably, other infections, like *Pneumocystis jirovecii* (PCP) pneumonia, may also present with elevated LDH and bilateral infiltrates, which may lead to lung injury and result in similar pulmonary complications (25). In this case, the patient was considered less likely to be co-infected with PCP due to the undetectable HIV viral load, a CD_4_ count >200 cells/mm^3^, CD_4_% above 14%, over 20 years on appropriate HAART treatment, and negative serum (1,3)-Beta-D-glucan in early admission. However, it is important to include PCP infection in the differential diagnosis and consider induced-sputum samples for a more definitive diagnosis, due to the similar laboratory and imaging findings in PCP and SARS-CoV-2 infections, and the rising number of reported coinfection cases (26).

Another proposed mechanism predisposing to spontaneous pneumothorax may also stem from the friability of the alveoli due to inflammation and persistent cough associated with SARS-COV-2 infection (24). Shirai et al. reported another case of bilateral spontaneous pneumothorax that developed later in the hospital course after receiving mechanical ventilation support, and was associated with bilateral pneumatoceles found on imaging (27). Our patient presented with bilateral apical pneumothoraces earlier in the hospitalization without initial mechanical ventilation or prior cystic findings on imaging. After the discovery of the left side tension pneumothorax, a chest tube was placed. However, later in the ICU admission, the patient's respiratory status declined and the patient required mechanical ventilation. Despite no underlying pulmonary conditions, the potential synergistic interaction between the history of HIV infection and SARS-CoV-2 infection may have impacted the inflammatory response and increased the risk for lung injury. Recently, the US Centers for Disease Control and Prevention has added HIV infection to the list of high risk conditions that predisposes adults to more severe SARS-CoV-2 infection (28). The potential mechanisms and clinical presentations remain to be studied (29). It is our hope that this case report raises awareness and promotes the investigation for the potential synergistic interaction between HIV and SARS-CoV-2 infections and the associated risk of pneumothorax development.

## Conclusion

This report emphasizes awareness of the possibility of spontaneous pneumothorax as a complication of SARS-CoV-2, especially in patients with a history of HIV infection. Early identification and management of pneumothorax may help reduce the associated morbidity and mortality and should be considered in the workup and treatment of a patient presenting with shortness of breath and suspected SARS-CoV-2 infection. To the best of our knowledge, this is the first case reporting bilateral spontaneous pneumothorax with later tension pneumothorax in a HIV-positive patient in the setting of SARS-CoV-2 infection. This patient's case also highlights the potential risk of rapidly developing a spontaneous pneumothorax in the setting of HIV and SARS-COV-2 infection, despite no prior history of respiratory disease, no active smoking, and no mechanical ventilation use. Importantly, the patient's comorbidity of HIV infection, although well-controlled, may have interacted with SARS-COV-2 in an inflammatory response that led to structural damage and resulted in a unique presentation of bilateral spontaneous pneumothoraces, and later complicated by a tension pneumothorax.

## Data Availability Statement

The datasets presented in this study can be found in online repositories. The names of the repository/repositories and accession number(s) can be found in the article/supplementary material.

## Ethics Statement

Ethical review and approval was not required for the study on human participants in accordance with the local legislation and institutional requirements. The patients/participants provided their written informed consent to participate in this study. Written informed consent was obtained from the individual(s) for the publication of any potentially identifiable images or data included in this article.

## Author Contributions

YC, SG, JA, JM, SM, and EM prepared the manuscript's first draft. YC, AH, and JK retrieved and corroborated the data. CK prepared the figures and edited the manuscript. LS-L, JA, SM, MH, CM, SZ, DG, AM-M, KH, KS, MS, RC, GA, LB, JK, AH, and EM collaborated in the discussion. All authors contributed to manuscript revision, read, and approved the submitted version.

## Conflict of Interest

The authors declare that the research was conducted in the absence of any commercial or financial relationships that could be construed as a potential conflict of interest.

## Publisher's Note

All claims expressed in this article are solely those of the authors and do not necessarily represent those of their affiliated organizations, or those of the publisher, the editors and the reviewers. Any product that may be evaluated in this article, or claim that may be made by its manufacturer, is not guaranteed or endorsed by the publisher.
